# Single-fraction radiation therapy in patients with metastatic Merkel cell carcinoma

**DOI:** 10.1002/cam4.458

**Published:** 2015-04-23

**Authors:** Jayasri G Iyer, Upendra Parvathaneni, Ted Gooley, Natalie J Miller, Elan Markowitz, Astrid Blom, Christopher W Lewis, Ryan F Doumani, Kaushik Parvathaneni, Austin Anderson, Amy Bestick, Jay Liao, Gabrielle Kane, Shailender Bhatia, Kelly Paulson, Paul Nghiem

**Affiliations:** 1 Department of Medicine/Dermatology, University of WashingtonSeattle, Washington; 2 Department of Radiation Oncology, University of WashingtonSeattle, Washington; 3 Fred Hutchinson Cancer Research CenterSeattle, Washington; 4 Seattle Cancer Care AllianceSeattle, Washington; 5 Department of Medicine/Oncology, University of WashingtonSeattle, Washington

**Keywords:** Merkel cell carcinoma, Merkel cell polyomavirus, single-fraction radiation therapy, skin cancer

## Abstract

Merkel cell carcinoma (MCC) is an aggressive, polyomavirus-associated cancer with limited therapeutic options for metastatic disease. Cytotoxic chemotherapy is associated with high response rates, but responses are seldom durable and toxicity is considerable. Here, we report our experience with palliative single-fraction radiotherapy (SFRT) in patients with metastatic MCC. We conducted retrospective analyses of safety and efficacy outcomes in patients that received SFRT (8 Gy) to MCC metastases between 2010 and 2013. Twenty-six patients were treated with SFRT to 93 MCC tumors located in diverse sites that included skin, lymph nodes, and visceral organs. Objective responses were observed in 94% of the measurable irradiated tumors (86/92). Complete responses were observed in 45% of tumors (including bulky tumors up to 16 cm). “In field” lesion control was durable with no progression in 77% (69/89) of treated tumors during median follow-up of 277 days among 16 living patients. Clinically significant toxicity was seen in only two patients who had transient side effects. An exploratory analysis suggested a higher rate of in-field progression in patients with an immunosuppressive comorbidity or prior recent chemotherapy versus those without (30% and 9%, respectively; *P* = 0.03). Use of SFRT in palliating MCC patients was associated with an excellent in field control rate and durable responses at treated sites, and with minimal toxicity. SFRT may represent a convenient and appealing alternative to systemic chemotherapy for palliation, for which most patients with oligometastatic MCC are eligible. SFRT may also synergize with emerging systemic immune stimulants by lowering tumor burden and enhancing presentation of viral/tumor antigens.

## Introduction

Merkel cell carcinoma (MCC) is an aggressive skin cancer with a 46% disease-associated 5-year mortality 1. Distant metastases are common (>30% of cases) and typically occur within 1–3 years following diagnosis 2. As the median age of MCC patients is ∼65 years, many patients are elderly with significant comorbidities. They are best managed by treatment that has minimal side effects, is convenient and cost-effective.

Traditional therapy for advanced metastatic MCC is cytotoxic chemotherapy or fractionated radiation. A small cell carcinoma chemotherapy regimen of carboplatin and etoposide is commonly used. Although most patients initially respond (reported response rate [RR] of 60%, 36% complete and 24% partial) 3, these responses are often not durable. Furthermore, chemotherapy is typically associated with significant side effects, and is limited to patients with good performance status. MCC is a radiosensitive cancer and fractionated radiotherapy (FRT), typically delivered at 30 Gy over 10 fractions, is often effective for MCC metastases. However, FRT is logistically inconvenient, requiring multiple visits to an RT center.

Cellular immunity plays a particularly important role in MCC survival. Multiple forms of systemic immune suppression have been linked with an increased incidence of MCC 4. Indeed, patients with systemic immune suppression have a significantly worse prognosis 5 independent of stage. Furthermore, the presence of intratumoral T-cell infiltration is associated in a stage-independent manner with improved MCC survival 6,7. Mouse model data suggest that single-fraction RT (SFRT) is more effective than FRT in augmenting local tumor immunity 8. A likely contributor to this observation may be that cytotoxic CD8 T-cells that are stimulated and recruited to the tumor following SFRT are not killed by subsequent RT fractions. Although SFRT (8 Gy) has been used safely for decades for the treatment of bone metastases in other cancers 9–11, there are no reports of the use of SFRT for MCC. Furthermore, there are only very limited data regarding SFRT for nonbone metastases (NBM) in other cancer types.

There was a pressing clinical need for palliative therapy in patients who were not candidates for fractionated radiation therapy due to logistical issues and who had developed lesions that were chemotherapy-resistant and symptomatic. Given the known safety of SFRT (8 Gy) for bone metastases of many cancer types, we began to treat patients with this approach in 2010. Here, we report a retrospective analysis of our experience treating advanced MCC metastases with SFRT. The data suggest significant benefit, excellent tolerability, and a link to intact cellular immunity for this approach.

## Patients and Methods

At initial evaluation, all patients were consented and enrolled into a FHCRC IRB-approved (#6585) prospective longitudinal database designed to assess outcomes relative to clinical features including stage and therapy.

### Inclusion criteria for study cohort

All patients with metastatic MCC that received 8 Gy SFRT, with a minimum follow-up of 6 weeks between 1 December 2010 and 15 February 2013 were included in this retrospective study. The treatment was offered to all MCC patients who presented to our center with oligometastatic disease (typically 1–5 lesions) and who had not previously received RT to the target lesion(s). Patients with more than a single lesion were treated to some or all of their lesions, depending on disease burden, necessity for palliation of particular lesions, proximity to other major organs or neurovascular regions, and other patient considerations. Patients could receive other systemic therapies concurrent with and subsequent to SFRT without being censored from the study. There were no anatomic locations that were deemed inappropriate for SFRT. The dataset was finalized on 13 May 2013, after which no new data were included. Bony lesions could not be included in analyses of measurable disease response because they cannot be assessed for size/RECIST responses by computed tomography scans. Therefore, eight target tumors in five patients with bone metastases were assessed separately for symptom relief goals.

### Treatment

SFRT (8 Gy) was delivered using electrons for skin and subcutaneous lesions, and photons using 3D conformal planning and IMRT (intensity modulated radiation treatment) for deeper tumors in the neck, mediastinum, and retroperitoneal regions. None of the patients were treated with stereotactic radiotherapy techniques. Standard doses of prophylactic antiemetic premedications including ondansetron and dexamethasone were routinely administered for 1–3 days beginning shortly before treatment of abdominal and retroperitoneal masses.

### Monitoring/evaluation

**Figure 1:**
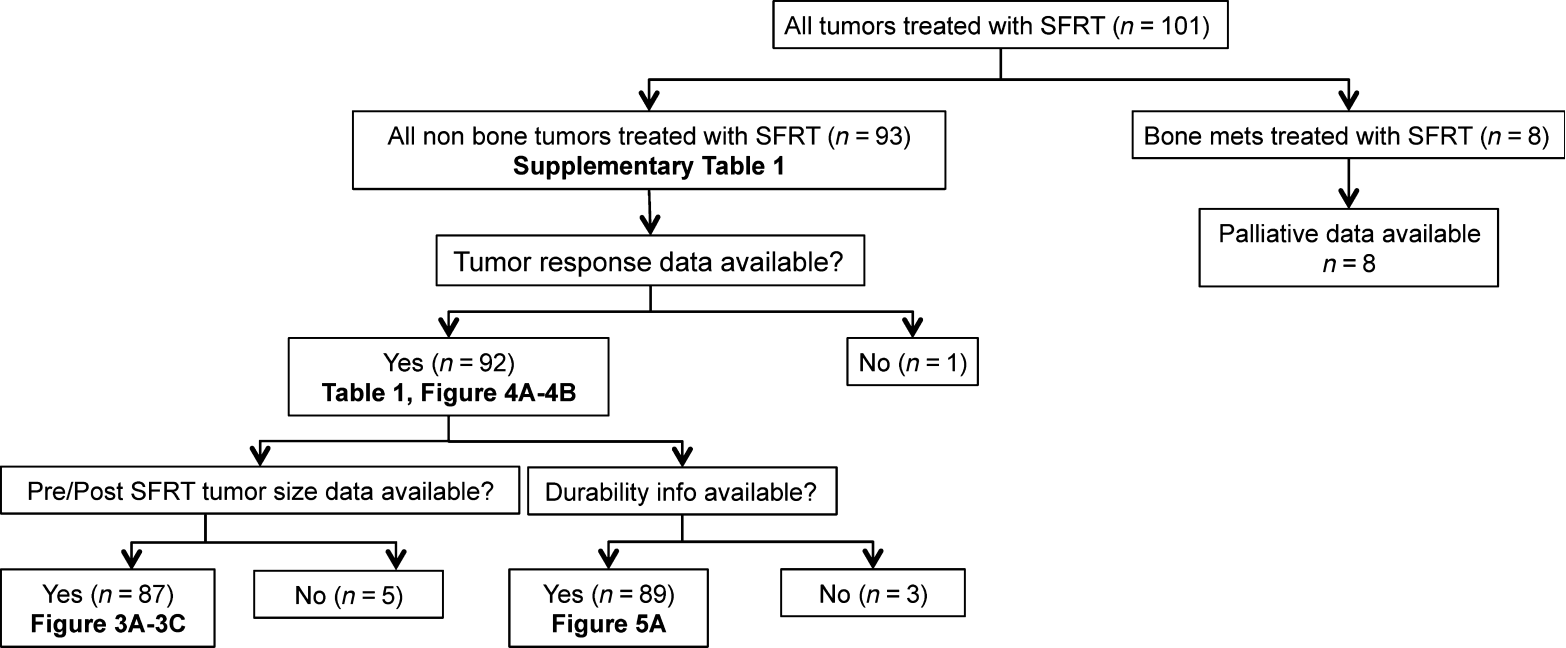
Flow diagram for 101 metastatic Merkel cell carcinoma lesions treated with single-fraction radiation therapy (SFRT). The diagram summarizes the available data used for the specified analyses and tables and figures in which those data are presented.

All patients (except those with superficial lesions treated with electrons) had a CT scan for RT planning and responses tracked via CT scan. Superficial lesions were tracked by measurement with a ruler, and/or digital photography. Toxicity was graded using the Common Terminology Criteria of Adverse Events v3.0. RTOG/EORTC late radiation morbidity scoring schema. Of the 93 tumors treated using SFRT, efficacy analysis was carried out on 92 tumors (see Fig.1). Response evaluation was by RECIST version 1.1 12, modified only in that pretreatment lesion size reported was the longest dimension of all tumors including lymph nodes. When lesions were grouped in extreme proximity, they were irradiated with a single-targeted dose of 8 Gy and their sizes were measured as a single lesion. Complete palliation for bone metastasis was defined as complete resolution of pain, which was the only presenting symptom for these patients.

### Data collection

For patients who received treatment at an outside facility following our initial assessment (of 93 tumors that received 8 Gy SFRT, 21 were treated at an outside facility) all treatment records including physician notes, dosimetry records, and scan data were obtained and analyzed. Baseline patient and tumor characteristics including number, size, location of treated lesions, immune status, exposure to previous, and subsequent treatments, RT date, response to treatment, acute and late toxicity were recorded until last follow-up, death of the patient, or the data collection was terminated for this study on 13 May 2013.

### Patient categories

Patients with chronic lymphocytic leukemia (CLL), HIV, those on immunosuppressive medications for solid organ transplant or autoimmune diseases, or exposed to cytotoxic chemotherapy for MCC prior to SFRT, were considered to have some degree of immunosuppression. We categorized patients into two categories, (1) low risk (LR) (patients with no known immune suppression or prior chemotherapy) and, (2) high risk (HR) (patients with known immune suppression or prior chemotherapy). Median time interval from chemotherapy to SFRT was 3.5 months (range 1.4–12.9 months).

### Statistical analysis

Responses were noted on a per-tumor basis rather than a per-patient basis as some patients had multiple tumors that were treated on one or more dates. The RR was defined as the number of tumors (individual metastases) with complete (CR) or partial responses (PR) divided by the total number of evaluable tumors. However, analyses comparing LR to HR patient groups were conducted by considering multiple tumors in some patients by using generalized estimating equations (GEE). This method appropriately adjusts the variance of estimated effects in order to take into account the fact that some patients have multiple tumors. A log-link function was used to estimate the odds ratio of response between groups. Durability of response was calculated as the interval between SFRT and treated lesion progression, last follow-up date, or death (if treated lesion never progressed). Statistical analyses were carried out with SAS software (version 9.3; Cary, NC).

### Survival curves

Survival that was free from progression of any treated lesion was estimated on a per-patient basis and was calculated as time between SFRT date and first progression of any treated lesion, death, or last follow-up (log-rank test was used to compare high- and low-risk patients). Durability of responses, tumor reduction percentage and magnitude of tumor responses were graphed using R statistical software, version 3.0.2 (R Foundation for Statistical Computing, Vienna, Austria) and ggplot2 (version 0.9.3.1, Hadley Wickham.).

## Results

### Patient and tumor characteristics

Between December 2010 and February 2013, 93 NBM in 26 patients were treated with SFRT. As shown in Table1, 85% of patients in this study were male and 15% were female. The median age at time of treatment was 68 years (range 54–96 years). Thirteen of 26 patients (50%) were classified as high-risk patients (those with known immune suppression and/or prior chemotherapy; 60 tumors) whereas 13 were low-risk (no known immune suppression or prior chemotherapy; 33 tumors). Among high-risk patients, one had immune suppression alone (three tumors), two had both immune suppression and prior chemotherapy (six tumors), and 10 had prior chemotherapy (53 tumors). Median tumor size among all patients was 4 cm (range: 1–19 cm) and the average number of tumors treated per patient was 3.5 (range 1–28). The median interval between first metastatic MCC diagnosis and SFRT for LR tumors was shorter than for HR tumors, likely because the initial treatment for metastatic disease was chemotherapy for the HR tumors, meaning that SFRT started later.

**Table 1:** Demographics of study cohort for RECIST-evaluable tumors

Patient characteristics	*N*
Number of patients	26
Sex	
Male	22
Female	4
Median age at time of treatment (range)	68 years (54–96)
Number of MCC metastases treated with 8Gy SFRT evaluable by RECIST	92
Low-risk patients (no. tumors)	13 (32)
High-risk patients (no. tumors)	13 (60)
Median tumor size (range)	4 cm (1–19)
Characteristics of HR patients	No. patients (tumors)
Immunosuppressive illness (myelodysplasia) + medication (chronic methotrexate)	1 (3)
Medications (chronic methotrexate, anti-rejection medications)	2 (4)
Immunosuppressive illness (CLL or myelodysplasia) + prior chemotherapy	2 (6)
Only prior chemotherapy	8 (47)
Median interval between MCC diagnosis and SFRT (range)	568 (24–1987)
Low-risk patient tumors	669 (56–1987)
High-risk patient tumors	413 (24–429)
Median interval between first metastatic MCC lesion and SFRT (range)	207 (9–813)
Low-risk patient tumors	113 (9–445)
High-risk patient tumors	366 (35–813)
Patient outcomes	
Median follow up time from SFRT among all living patients (range)	277 days (104–699)
Low-risk patients	277 days (104–499)
High-risk patients	256 days (175–699)
Median time to treated lesion progression in days (no. of treated tumors that progressed)	
Low-risk tumors (3 of 32)	193 days
High-risk tumors (17 of 57) 1	71 days

1 Among 60 high-risk tumors, treated lesion progression data was available only for 57 tumors. For remaining 3 tumors treated lesion progression was unknown.

### Efficacy

**Figure 2:**
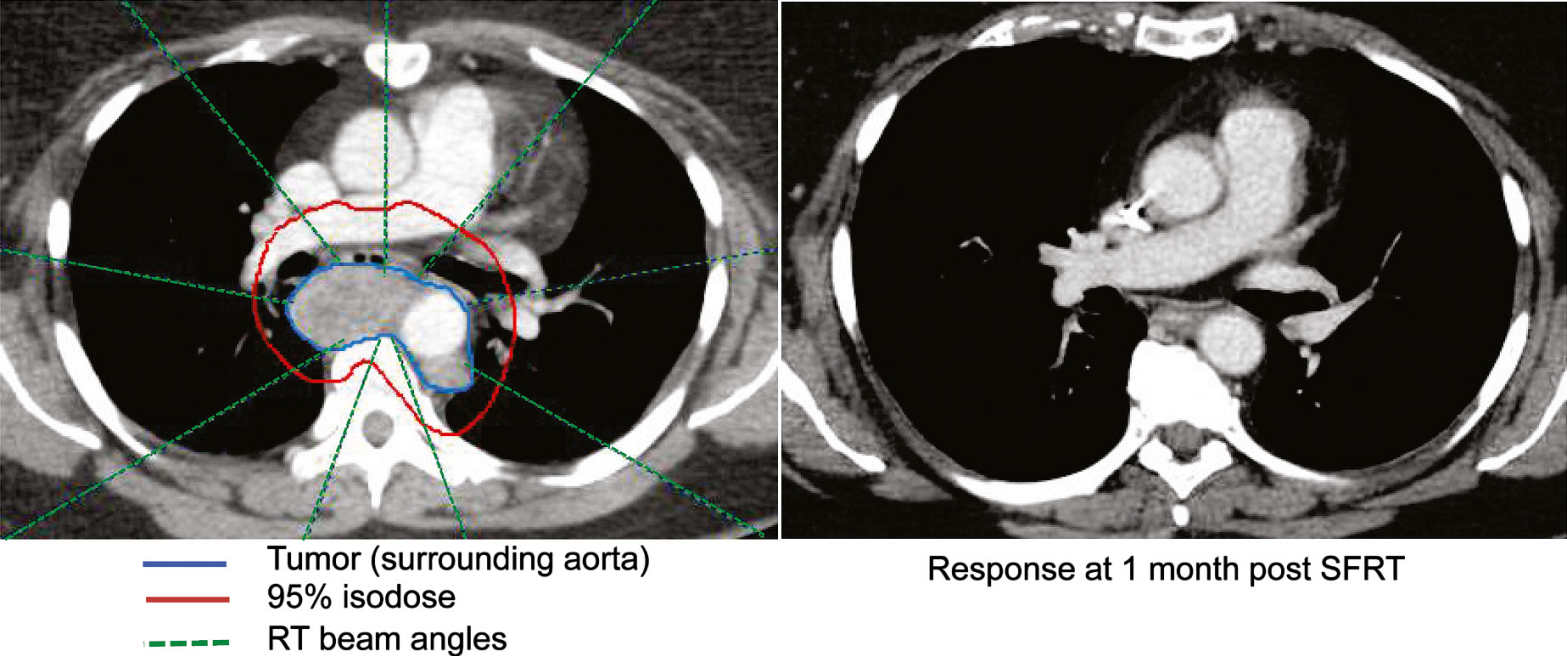
Radiotherapy plan and tumor response 1 month after SFRT. Left panel: A 56-year-old woman with recent stage IIIb MCC developed shortness of breath associated with a subcarinal paraesophageal lymph node metastasis (tumor outlined in blue, surrounding the aorta which is contrast-enhanced). She underwent SFRT, experienced no side effects from therapy, had full resolution of symptoms by day 5 after treatment, and by 1 month had a complete response as documented by CT scan (right panel). The red line represents the RT dose covering the tumor and the green dashed lines depict the nine RT beam angles directed at the tumor. The 95% isodose line in the radiotherapy plan closely conforms to the treated tumor in three dimensions, and dose was minimized to surrounding critical structures including spinal cord, heart and lungs. This tumor is included as lesion #23 in Table S1, and had not recurred as of the end of study period (11 months) or at last follow-up (22 months after SFRT). SFRT, single-fraction radiation therapy; MCC, Merkel cell carcinoma.

**Figure 3:**
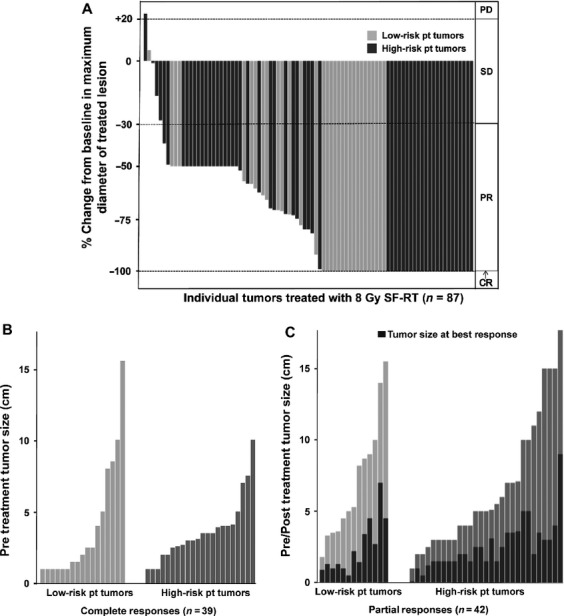
Tumor responses to SFRT: Of 92 tumors, 87 had both pre- and post-SFRT size measurements and could be included in this analysis (summarized in Fig.1). In each panel, light gray bars represent low-risk patients who have no known immunosuppression and have not received prior chemotherapy; dark gray bars represent high-risk patients who have known systemic immune suppression and/or have received prior chemotherapy for MCC. (A) A waterfall plot of the percent change in largest treated lesion diameter at best response after SFRT as compared with baseline. Response criteria as per RECIST 1.1 12 are as indicated on right of graph: CR, complete response; PR, partial response; SD, stable disease; PD, progressive disease. (B) The pretreatment tumor size (largest dimension, in cm) for treated lesions that had a CR. 39 tumors with pretreatment measurements (22 high risk and 17 low risk) achieved CR. (C) The reduction in tumor size comparing pretreatment to best response for treated lesions that had a PR. Forty-two tumors (29 high risk and 13 low risk) achieved PR. The black bars in (C) (tumors with partial response) indicate tumor size at best response for each tumor. SFRT, single-fraction radiation therapy; MCC, Merkel cell carcinoma.

**Figure 4:**
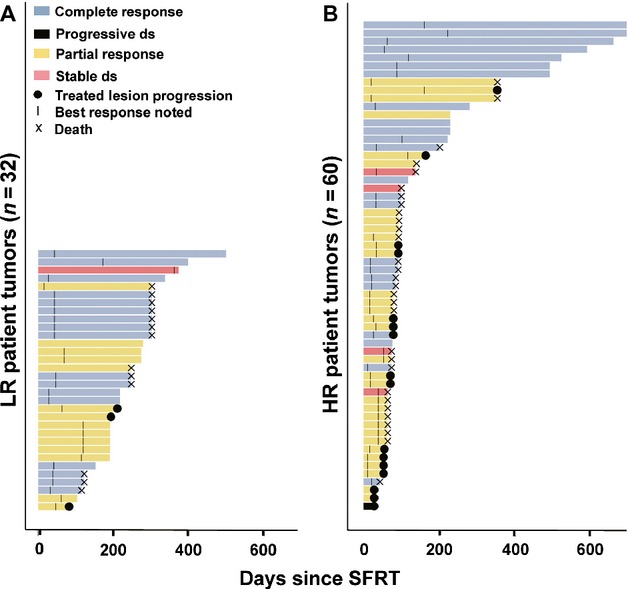
Durability of tumor responses. The period during which each treated tumor could be evaluated is plotted as a function of time in days since single-fraction radiation therapy (SFRT). “Events” were noted using symbols defined in the key at top left. Notably, none of the tumors that had a complete response (light blue bars) ever recurred. Tumors that have no symbol at the right side of their bar were not associated with progression or death at the time of last follow-up. (A) Represents tumors from low-risk patients that were treated with SFRT. (B) Represents tumors from high-risk patients treated with SFRT.

A representative intensity modulated radiation therapy (IMRT) plan for targeting a mediastinal metastasis is shown in Figure2. The posttreatment CT scan demonstrated complete resolution of the tumor, which was durable throughout the study period. Ninety-four percent of tumors responded (CR or PR) to SFRT. Five lesions were stable in size after SFRT, and one progressed (Fig.3A). Although a higher fraction of treated tumors in LR patients had a CR (53%; 17 of 32) than of tumors in HR patients (37%; 22 of 60) this difference was not statistically significant (*P* = 0.51, GEE). As shown in Figure3B, the size distribution for tumors that achieved CR or PR was similar. For lesions that could be assessed clinically (symptomatic and or superficial lesions) responses were typically noted by 7–10 days after therapy. For lesions requiring CT scan assessment, responses were usually seen at the first study following therapy (see Fig.4). We did not observe spontaneous shrinkage of nontreated MCC tumors following SFRT (abscopal effect) in any of the cases during the study period. However, in the majority of patients we treated all the presenting lesions negating the ability to observe for potential abscopal effects.

### Durability of responses

**Figure 5:**
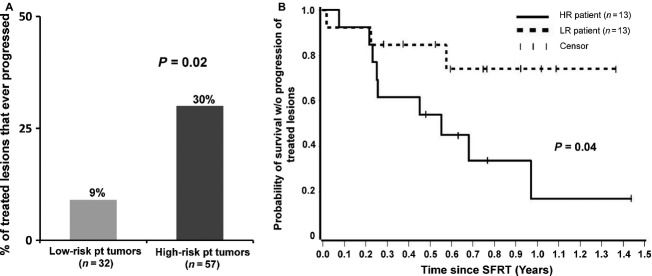
Risk of disease progression. (A) Risk of progression of single-fraction radiation therapy (SFRT)-treated lesions. 9% of tumors (three of 32) in low-risk patients progressed as compared to 30% of tumors (17 of 57) in high-risk patients (*P* = 0.02). (B) Survival without progression of treated lesions. The fraction of patients who were alive and remained free of progression from SFRT-treated lesion(s) is plotted as a function of years after SFRT.

Eighty-nine tumors treated with SFRT also had data allowing assessment of durability beyond best response (Fig.1). Sixty-nine of the 89 lesions (77%) did not progress during median follow-up of 8.4 months among living patients. CRs were durable, as none of the 40 tumors that achieved a CR recurred, regardless of the HR or LR status of the patient. Among the 20 lesions that progressed during the study period, the median time to treated lesion progression following SFRT was 2.5 months. Most strikingly, only 9% (three of 32) of tumors from patients in the LR group ever progressed at the treated site, as compared with 30% (17 of 57) for tumors in patients in the HR group (odds ratio: 0.24, *P* = 0.02, 95% CI, 0.07–0.81, GEE*;* Fig.5A). Furthermore, among tumors that ultimately progressed, the interval between SFRT and progression was longer for tumors arising in LR patients (Table1; 193 days) as compared to high-risk patients (71 days).

### Patient outcome

During the study period, two of 13 patients who were in the LR category died of MCC and one patient in this category died of an unknown cause, most likely not MCC (96-year-old man without evidence of MCC at time of death). In contrast, seven of 13 HR patients died of MCC during the study period. There were no deaths within 6 weeks of SFRT in either group. Median follow-up from first SFRT to last contact among the 16 surviving patients was 277 days (range, 104–699 days). Among the 10 patients who died, the time from SFRT to death ranged from 2.8 to 13.0 months with a median of 6.4 months. Survival free from progression of any treated lesion was significantly greater in LR patients than in HR patients (*P* = 0.04, log-rank test) and is plotted in Figure5B.

### Palliative efficacy for bone metastases

Patients had complete resolution of pain for 5/8 bone metastases (63%) treated with SFRT and the remaining three bone metastases had marked, but incomplete elimination of pain. All five complete palliation responses were durable throughout the study period.

### Adverse events

No side effects of SFRT were noted in 24 of 26 patients, supporting a high degree of tolerability for the SFRT approach. The two patients who experienced side effects received therapy for large tumor volumes. Specifically, one patient underwent treatment of a 15 × 11 × 11 cm abdominal mass. He developed nausea and vomiting following SFRT that lasted 72 h and required hospitalization for IV hydration and antiemetic therapy. He had an excellent tumor response and did not require further treatment for over 10 months. Another patient who underwent simultaneous treatment of multiple subcutaneous, inflamed tumors developed a “flare pain” reaction that lasted <4 h. The patient presented to an emergency room and was successfully managed with nonsteroidal anti-inflammatory medication. There were no late/long-term effects attributable to SFRT.

## Discussion

MCC is an aggressive, polyomavirus-associated skin cancer that is typically very radiosensitive. Development of metastatic MCC occurs in >30% of patients, however, options for treating metastatic disease are limited and unsatisfactory. In this retrospective study, we found a high RR (94%), excellent tolerability, and durable palliation for metastatic MCC lesions treated with SFRT. Indeed, objective responses were high among all MCC patients and durability of tumor responses was improved among patients without an immunosuppressive comorbidity or prior recent chemotherapy (low-risk patient group).

SFRT has been compared to fractionated RT for bone metastases in other cancers where it has been found to be safe and effective in the palliative setting 10,11,13,14. In a multicenter randomized study, Badzio et al. compared the efficacy of 4 Gy × 5 fractions with 8 Gy × 1 fraction for palliative therapy in bone metastases of breast, kidney, lung, prostate, and other cancers, and found that both treatments were equally effective 15. Hoskin et al. 11 and Jeremic et al. 14 investigated the optimal SFRT dose by comparing results from 4, 6, and 8 Gy SFRT for bone metastasis from primary breast, prostate, thyroid, lung, kidney cancers, and myeloma. They found that the overall response rate in patients treated with 6 Gy (73%) and 8 Gy (78%) was significantly better than the response rate for patients treated with 4 Gy (59%), and that patients treated with 6 or 8 Gy achieved faster onset of pan relief than those that received 4 Gy. In our study, 94% of MCC tumors demonstrated a response to 8 Gy SFRT. This RR is higher than the 60–70% reported for bone metastasis 16. However, this could be partially due to differences in the response evaluation for bone metastases versus the RECIST criteria used in our study. In addition, the higher RR could be reflective of the intrinsic radiosensitivity of MCC compared to other epithelial tumors (e.g., breast, lung, prostate, bowel, etc.) treated in the bone metastases studies.

While RRs of bone metastases to SFRT and FRT are comparable, data from randomized trials indicate responses are more durable following FRT 10. For example, although a meta-analysis by Wu et al. 16 reported similar RRs, retreatment was more frequent in patients that received SFRT (11–25%) as compared to FRT (0–12%) 16. It is possible that the more durable palliative effect of FRT in bone metastases could be due to the significantly higher overall dose of 30 Gy in FRT, compared to 8 Gy in SFRT. In this study, we did not compare SFRT and FRT responses. Among patients who received SFRT, we found that responses (and symptom relief) were rapid among all patients but significantly more durable for the low-risk group than the high-risk group (Fig.5A). There was no progression of tumors that achieved a CR in either patient group at the end of the study period (median follow-up of 7.6 months). It is plausible that in our study, the rapid initial responses typically seen in both high- and low-risk groups was due to the direct effect of RT on the tumor, independent of the immune response. In contrast, the improved durability of responses in the low-risk group may be due to the presence of a more functional immune system.

We hypothesized that SFRT might augment cellular immunity, a particularly important feature for control of MCC 6,17. There is substantial evidence that RT is capable of converting the irradiated tumor into an immunogenic hub. Animal studies suggest that low dose (2–4 Gy) SFRT can promote tumor immunity via major histocompatibility complex (MHC) up-regulation, antigen presentation, and vascular normalization 18. At higher doses, SFRT likely retains these immunogenic effects, but also recruits T cells into the tumor and leads to greater direct tumor cell death due to apoptosis or necrosis 18. Using a B16 mouse melanoma model, Lee et al. showed that SFRT (20 Gy) is more effective than fractionated radiation therapy (FRT; 45 Gy in 3 fractions) in controlling tumors though the total dose of radiation was far less 8. In their model, the efficacy of SFRT was dependent on CD8 T-cells.

As an exploratory analysis, to determine whether a patient’s immune status might have had bearing on the efficacy of SFRT, we segregated our cohort into two groups: low-risk (no apparent immune suppression or prior chemotherapy) and high-risk (known immune suppression and/or prior chemotherapy). Due to the size of our cohort (26 patients), the study lacked sufficient power to separately analyze patients who had prior chemotherapy as compared to those with other types of immune suppression. In addition, several patients had both risk factors. We thus combined patients with any form of immune suppression into one high-risk group. Indeed, several prior studies have demonstrated that chemotherapy can cause clinically significant and persistent T-cell immune suppression 19–21. One study of 213 patients who received cytotoxic chemotherapy found that T-cell function was not normalized 12 months post chemotherapy 22. In our cohort, the median time interval from chemotherapy to SFRT was 3.5 months (range 1.4 - 12.9 months), well within the documented interval for persistent T-cell suppression following chemotherapy. The patient receiving SFRT 12.9 months after chemotherapy also received SFRT at 9.4 and 10.4 months after chemotherapy and was thus classified as high risk. It is likely that other factors, besides immune function, could have contributed to the poorer outcomes in our high-risk patient cohort. For example, it is plausible that prior chemotherapy selected for radio-resistant tumor populations.

Although the vast majority of tumors in both groups responded, the durability of responses of treated lesions was significantly improved in low-risk patients (Fig.5A). Although there are other possible explanations as noted above, the improved durability of responses in the low-risk group is analogous to the prolonged disease control seen with immune-based therapies for melanoma 23.

Although 94% of SFRT-treated tumors responded, we did not note spontaneous distant disease regression (abscopal effect) during the study period in any patient. There is evidence in a preclinical model that optimal dosing of radiation for inducing a systemic immune effect (compared to local effects studied by Lee et al.) may require more than a single fraction. Dewan et al. compared the efficacy of three RT regimens: 20 Gy × 1 fraction, 8 Gy × 3, and 6 Gy × 5 in combination with anti-CTLA4 (the latter had no effect on either model when given alone) and concluded that three or five radiation fractions provided a greater immune-stimulating effect at distant, nonradiated sites as compared to a single fraction of radiation 24.

There are several limitations of this study. Because this was a retrospective analysis, it is possible that inadvertent biases relating to patient selection, tumor response assessment or treatment techniques could have affected the results. In terms of patient selection, SFRT was offered to all patients with oligometastatic disease. The number of patients with immunosuppression not due to chemotherapy was limited and hence we were unable to separately analyze immune suppression in the absence of chemotherapy versus chemotherapy alone. There was variability in the timing of posttherapy evaluation of tumor responses. However, we do not believe that this factor would be likely to introduce systematic bias to the results. Regarding variability of treatment techniques, the majority (76%) of tumors were treated at our facility, minimizing interfacility variation. Moreover, patients treated elsewhere had results that were analogous to those from our own facility (94% response at our institution compared to 95% at outside institutions). Additionally, our cohort included patients with high disease burden (multiple or bulky tumors) in whom no single treatment modality was sufficient to control disease. Specifically, the majority (15/26) of patients treated with SFRT received one or more other systemic treatment modality concurrent with (four patients) or subsequent to (11 patients) SFRT that could have affected the efficacy of SFRT. These treatments included anti-CD137 antibody (three patients), pazopanib (six patients), somatostatin receptor analog (four patients), T-cell therapy (one patient), anti-PD1 (one patient), and cytotoxic chemotherapy (five patients). The median time between SFRT and initiation of subsequent systemic therapy in these 11 patients was 37 days. However, clinicians typically noted responses to SFRT within 7–10 days of treatment. In summary, because responses to SFRT were typically noted before initiation of systemic therapy, and because lesions in patients who received subsequent systemic therapy progressed at a similar rate (29%) to the entire group (23%), we believe that the efficacy observed at treated sites was likely due to SFRT rather than to other regimens.

This study demonstrates the safety and efficacy of SFRT for a wide range of metastatic MCC tumor locations. In a population with advanced age and comorbidities, the lack of toxicity and convenience of a single treatment approach is noteworthy. This study also demonstrates that MCC patients that have apparently normal immune status (low-risk) have significantly better response durability compared to those with known immune compromise and/or recent chemotherapy (high risk). This reinforces observations in MCC that strongly link immune status with disease control 6,7. In order to improve durability of response for the high-risk patients, it may be indicated to explore RT dose escalation and/or a modest increase in number of fractions. Further studies that correlate the immune status of patients with the immune milieu of the tumor microenvironment should be carried out to identify differences between tumors that respond and those that do not. Such studies may also suggest strategies to augment antitumor immunity in unresponsive tumors. SFRT could be combined with emerging systemic immune stimulants such as immune checkpoint inhibitors to improve outcomes for this aggressive disease by lowering tumor burden and exposing viral/tumor antigens.

## Supporting Information

**Table S1.** Tumor characteristics (^myelodysplasia, *MTX for rheumatoid arthritis, ^+^Kidney transplant).
