# Supplementary material for: Single-fraction radiation therapy in patients with metastatic Merkel cell carcinoma
Source: Cancer Med. 2015 Apr 23;4(8):1161–70. doi: 10.1002/cam4.458 (PMC4559027; doi:10.1002/cam4.458)
Supplement: Supplementary file 1 [file cam40004-1161-sd1.docx]

**Supplemental Table: Tumor Characteristics (**^myelodysplasia, *MTX for rheumatoid arthritis, ^+^Kidney transplant**)**

| **Pt ID** | **Age** | **Sex** | **Immune**  **suppressed** | **Prior chemo** | **Lesion**  **ID** | **Tumor Site** | **Tumor size**  **(cm)** | **Response** | **Treated lesion**  **Progression (days)** | **Status** |
| --- | --- | --- | --- | --- | --- | --- | --- | --- | --- | --- |
| 1 | 73 | F | Y^^,^* | N | 1 | R thigh | 2.5 | CR | NA | A |
|  |  |  |  |  | 2 | R upper calf | 1 | CR | NA |  |
|  |  |  |  |  | 3 | R lower calf | 1 | CR | NA |  |
| 2 | 66 | M | N | N | 4 | Pancreas, porta hepatis | 8.5 | CR | NA | A |
| 3 | 56 | M | N | Y | 5 | Paraaortic/ Retrocrural LN | 4.1 | CR | NA | A |
|  |  |  |  |  | 6 | L iliopsoas mass | 7.1 | PR | 355 |  |
|  |  |  |  |  | 7 | L paracolic mass | 3.9 | CR | NA |  |
|  |  |  |  |  | 8 | L submandibular gland | 2.6 | CR | NA |  |
|  |  |  |  |  | 9 | L splenic flexure mass | 3.1 | CR | NA |  |
| 4 | 68 | M | N | N | 10 | Central para aortic mass | 8 | CR | NA | A |
| 5 | 60 | M | N | N | 11 | Pregastric LN | 2 | SD | NA | A |
| 6 | 71 | M | N | Y | 12 | L face and L neck | 2 | CR | NA | A |
| 7 | 55 | M | N | N | 13 | Paraaortic pancreatic/ Retrocrural LN | 14 | PR | NA | A |
| 8 | 68 | M | N | Y | 14 | Chest subcarinal mass > 5cm, liver, adrenal | 7 | CR | NA | D |
| 9 | 74 | M | N | N | 15 | L upper arm | 2.5 | CR | NA | A |
|  |  |  |  |  | 16 | L lower arm | 2.5 | CR | NA |  |
| 10 | 72 | M | N | Y | 17 | Liver | 5.5 | PR | NA | A |
|  |  |  |  |  | 18 | R neck LN | 1 | CR | NA |  |
|  |  |  |  |  | 19 | L neck LN | 3.5 | CR | NA |  |
|  |  |  |  |  | 20 | R neck LN | 2.7 | CR | NA |  |
|  |  |  |  |  | 21 | R anterior neck LN (under R ear) | 3.5 | CR | NA |  |
| 11 | 70 | M | Y* | N | 22 | L para/infrarenal & paraaortic LN | 7.5 | CR | NA | A |
| 12 | 56 | F | N | N | 23 | Paraesophageal LN | 15.5 | CR | NA | A |
| 13 | 76 | M | N | N | 24 | L axillary LN | 1 | CR | NA | A |
| 14 | 65 | M | N | Y | 25 | Pancreatic tail | 5.1 | PR | 165 | A |
| 15 | 85 | M | N | N | 26 | LN adjacent to IVC | 3.6 | PR | NA | A |
|  |  |  |  |  | 27 | LN superomedial/adjacent to the R diaphragm pericaval LN | 3.3 | PR | NA | A |
|  |  |  |  |  | 28 | Lateral aspect of R hepatic lobe | 5.3 | PR | NA | A |
| 16 | 54 | F | N | N | 29 | R groin mass | 4.5 | PR | NA | A |
|  |  |  |  |  | 30 | R obturator LN | 8.7 | PR | NA |  |
|  |  |  |  |  | 31 | R common iliac LN | 1.8 | PR | NA |  |
|  |  |  |  |  | 32 | Paraortic LN | 8.2 | PR | NA |  |
| 17 | 60 | M | N | N | 33 | Mass abutting L kidney | 15.5 | PR | NA | A |
| 18 | 96 | M | N | N | 34 | R axilla/SCF/low neck | 9 | PR | 82 | D |
| 19 | 59 | M | Y* | N | 35 | Nodes – R pelvic/groin | 19 | PR | Unknown | D |
|  |  |  |  |  | 36 | Popliteal fossa | 6 | PR | Unknown |  |
|  |  |  |  |  | 37 | Porta-hepatis, paracaval and interaortocaval LN | Unknown | PR | Unknown |  |
| 20 | 68 | M | N | Y | 38 | R lung apex | Unknown | CR | NA | D |
|  |  |  |  |  | 39 | Pancreatic head and tail | 5 | CR | NA |  |
|  |  |  |  |  | 40 | R & L testicle | 4 | CR | NA |  |
| 21 | 67 | M | N | N | 41 | Paraaortic, peripancreatic LN | 10 | PR | 210 | D |
| 22 | 78 | M | Y+ | Y | 42 | R preauricular LN | 2 | PR | NA | D |
|  |  |  |  |  | 43 | R neck LN near mandible | 3 | PR | NA |  |
|  |  |  |  |  | 44 | Scalp | 5 | PR | NA |  |
| 23 | 72 | M | Y^ | Y | 45 | R axillary LN | 2 | PR | 28 | D |
|  |  |  |  |  | 46 | Chest wall | 1 | PR | 28 |  |
|  |  |  |  |  | 47 | Liver R posterolateral segment | 4 | PD | 28 |  |
| 24 | 76 | F | N | Y | 48 | R shoulder | 10 | CR | 79 | D |
|  |  |  |  |  | 49 | R scapula | 11 | PR | 79 |  |
|  |  |  |  |  | 50 | R kidney hilum | 6 | PR | 79 |  |
|  |  |  |  |  | 51 | L kidney hilum | 3 | CR | NA |  |
|  |  |  |  |  | 52 | L paravertebral | 2 | CR | NA |  |
|  |  |  |  |  | 53 | L ant abdominal subcutaneous lesion | 4 | SD | NA |  |
|  |  |  |  |  | 54 | L entire Breast | 18 | PR | NA |  |
|  |  |  |  |  | 55 | L upper back | 15 | PR | 71 |  |
|  |  |  |  |  | 56 | R upper back | 15 | PR | 71 |  |
|  |  |  |  |  | 57 | L axillary LN | 4 | CR | NA |  |
|  |  |  |  |  | 58 | L axillary LN | 3.5 | CR | NA |  |
|  |  |  |  |  | 59 | R breast UQ | 10 | PR | NA |  |
|  |  |  |  |  | 60 | R axilla | 4 | PR | NA |  |
|  |  |  |  |  | 61 | R shoulder | 10 | PR | 55 |  |
|  |  |  |  |  | 62 | R upper chest wall | 7 | PR | 55 |  |
|  |  |  |  |  | 63 | R flank | 4 | SD | NA |  |
|  |  |  |  |  | 64 | L flank | 5 | PR | NA |  |
|  |  |  |  |  | 65 | R mid back | 7 | PR | 53 |  |
|  |  |  |  |  | 66 | L mid back | 12 | PR | 53 |  |
|  |  |  |  |  | 67 | L lateral back | 15 | PR | 53 |  |
|  |  |  |  |  | 68 | L upper back | 3 | CR | NA |  |
|  |  |  |  |  | 69 | Sternum lesion 1 | 3 | PR | NA |  |
|  |  |  |  |  | 70 | Sternum lesion 2 | 2.5 | PR | NA |  |
|  |  |  |  |  | 71 | L anterior upper arm | 4 | PR | NA |  |
|  |  |  |  |  | 72 | L posterior upper arm | 3 | PR | NA |  |
|  |  |  |  |  | 73 | mid abdominal skin lesion | 4 | PR | NA |  |
|  |  |  |  |  | 74 | R abdominal skin lesion | 3 | SD | NA |  |
|  |  |  |  |  | 75 | L anterior chest lesion | 3 | PR | NA |  |
| 25 | 67 | M | N | Y | 76 | Tongue | 5 | PR | 92 | D |
|  |  |  |  |  | 77 | R upper neck | 3 | PR | 92 |  |
|  |  |  |  |  | 78 | Thyroid + lower neck | 8.5 | SD | NA |  |
| 26 | 85 | M | N | N | 79 | L side of penis ulcerated with heaped up nodularity | 5 | CR | NA | D |
|  |  |  |  |  | 80 | R side of penis | 4 | CR | NA |  |
|  |  |  |  |  | 81 | Sup suprapubic | 2 | CR | NA |  |
|  |  |  |  |  | 82 | Inferior suprapubic | 10 | CR | NA |  |
|  |  |  |  |  | 83 | Medial low back | 1.5 | CR | NA |  |
|  |  |  |  |  | 84 | Lateral low back | 1 | CR | NA |  |
|  |  |  |  |  | 85 | L inguinal/thigh lesion | 3.5 | PR | NA |  |
|  |  |  |  |  | 86 | L groin mass | 5 | PR | 193 |  |
|  |  |  |  |  | 87 | L penile tip | 1 | PR | NA |  |
|  |  |  |  |  | 88 | Dorsal sup glans | 1 | CR | NA |  |
|  |  |  |  |  | 89 | Sup distal thigh | 1.5 | CR | NA |  |
|  |  |  |  |  | 90 | R scrotum | 1 | Unknown | Unknown |  |
|  |  |  |  |  | 91 | Low back skin metastasis | 1 | CR | NA |  |
|  |  |  |  |  | 92 | L upper groin fold nodule | 1 | CR | NA |  |
|  |  |  |  |  | 93 | L groin nodule | 1 | CR | NA |  |
